# Amenability of the Gatekeeper Enzyme HphA to Engineering in the Homologation Pathway of l‑Phenylalanine and l‑Tyrosine through Homology-Based Site-Directed Mutagenesis

**DOI:** 10.1021/acsomega.5c12112

**Published:** 2026-02-17

**Authors:** Rebecca M. Lang Harman, H. Grace Blackstone, Favor O. Aruna, Shivam R. Patel, Minho Shin, Reed K. NeSmith, D. Brooks Dickson, Angela C. Spencer, Shogo Mori

**Affiliations:** † Department of Chemistry and Biochemistry, College of Science and Mathematics, 1421 Augusta University, Augusta, Georgia 30912, United States; ‡ Department of Biological Sciences, College of Science and Mathematics, 1421 Augusta University, Augusta, Georgia 30912, United States

## Abstract

Homologation of amino acids, the addition or deletion of a methylene group onto their side chains, has the potential to increase the biostability and bioavailability of peptide natural products. The first enzyme in the homologation pathway, HphA, was previously characterized and is substrate selective. Bioinformatics studies were used to identify amino acids in the active site of HphA that may play a role in substrate selection, by comparison to homologous enzymes, homocitrate synthase (HCS) and 2-isopropylmalate synthase (IPMS). Single-point mutants for five amino acid residues in HphA’s active site were created to mimic those found in HCS and IPMS. Their activities were measured via time-course assays with the natural substrates for HCS and IPMS. Residue A73 was identified as important for the substrate specificity of HphA; therefore, six additional mutations were generated and tested with nine substrates with various side chains. The HphA A73L mutant exhibited the highest activity compared to the other mutants, showing activity with counterparts of l-Tyr (HphA natural substrate), l-Val (IPMS natural substrate), l-Leu, l-Ser, l-Trp, and l-Asp. Kinetic assays were performed with HphA A73L using the active substrates and compared with kinetic data from HphA WT, HCS, and IPMS. These results demonstrated that the A73L mutation significantly relaxed the substrate specificity of HphA, indicating its amenability to engineering. This research will serve as the foundation for future metabolic engineering studies of the enzymatic homologation pathway of amino acids.

## Graphical abstract

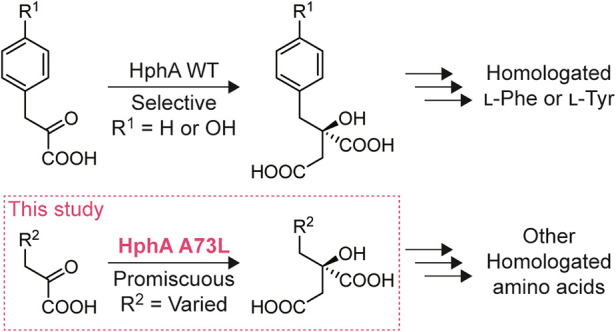

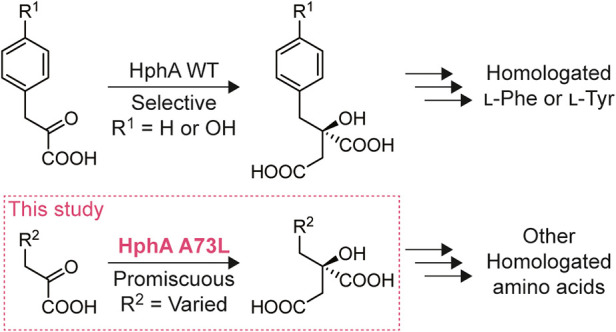

## Introduction

Natural products (NPs) are compounds found in nature that are synthesized by living organisms.^1^ These substances are characterized by their high molecular weight and structural complexity, properties that may be difficult to achieve via traditional organic chemistry methods.^2^ These features make NPs an essential component in the development of small molecule drugs.^3^ The medicinally useful NPs include peptide NPs due to their high diversity and biological activities.^4^ Most of them are categorized as either nonribosomal peptides (NRPs) or ribosomally synthesized and post-translationally modified peptides (RiPPs). RiPPs are synthesized using only canonical amino acids but are extensively post-translationally modified to produce diverse structures.^5^ NRPs exhibit significant structural diversity, with many containing nonproteinogenic amino acids, which enhance the biostability and bioavailability of peptide natural products.^6^

Homologation of amino acids is the insertion or deletion of a methylene group in the side chain of an amino acid (Figure 1), generating a nonproteinogenic amino acid in a peptide NP. It is hypothesized that this modification increases the stability and bioavailability of the peptide NP.^7^ One of the common NP families containing homologated amino acids (homoAAs) is the anabaenopeptins, which are produced by a variety of cyanobacteria.^8^ The genes responsible for the homologation of l-Phe and l-Tyr in anabaenopeptin biosynthesis in the cyanobacterium *Nostoc punctiforme* PCC 73102, *hphA/B/CD*, were previously identified, also noting the high substrate selectivity of the homologation pathway.^9^ The homologation of l-Phe and l-Tyr begins with the enzyme aromatic amino acid aminotransferase (ArAT). This is followed by the synthesis of a maleic acid derivative that incorporates the side chains of l-Phe and l-Tyr, facilitated by the enzyme 2-benzylmalate synthase (HphA). The resulting product undergoes several modifications through the action of 3-benzylmalate dehydratase (an isomerase; HphCD), benzylmalate dehydrogenase (HphB), and ArAT, leading to the formation of the homologated forms of l-Phe and l-Tyr.^9^ It is important to note that some aminotransferases (ATs) are known for their relaxed specificity^10^ and are typically encoded outside the relevant gene cluster for primary metabolism,^8^ with some exceptions.^11^ Previous metabolic engineering studies on the homologous pathways in bacterial and plant primary metabolism for l-Val^12^ and l-Met^13^ homologations, respectively, suggested that the first enzyme in the pathway, HphA, acts as the gatekeeper. Indeed, HphA was recently characterized as a highly selective enzyme.^14^ While modifying existing NPs through known homologation pathways would be advantageous, the high substrate selectivity of HphA remains a significant obstacle.

**Figure 1:**
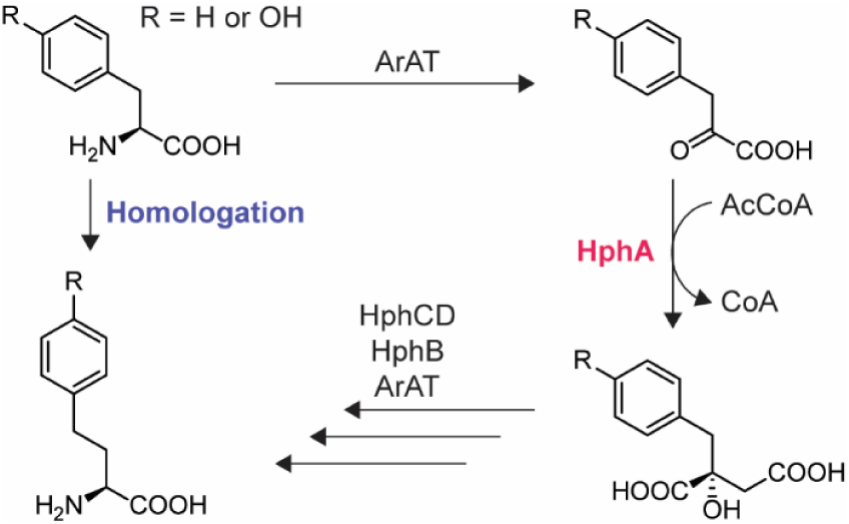
Homologation pathway for l-Phe and l-Tyr, focusing on the target enzyme HphA in this study. ArAT = aromatic amino acid aminotransferase, AcCoA = acetyl coenzyme A, CoA = coenzyme A.

The goal of this study was to determine whether HphA is amenable to engineering. Bioinformatics was used to design mutants to mimic the activity of its homologous enzymes, homocitrate synthase (HCS) and 2-isopropylmalate synthase (IPMS) for l-Lys and l-Leu biosynthesis, respectively. Site-directed mutagenesis was used to insert the mutations, and the activities of the mutant enzymes were characterized using colorimetric assays with Ellman’s reagent.^15^ The amino acid residue in the HphA active site responsible for the selection of the substrate was identified; the amenability of HphA to engineering was demonstrated by relaxing its substrate selectivity.

## Materials and Methods

### Bacterial Strains, Plasmids, Materials, and Instrumentation

Chemically competent *Escherichia coli* DH5α and BL21­(DE3) cells were purchased from Thermo Scientific (Waltham, MA) and Invitrogen (Thermo Scientific), respectively. DNA oligonucleotides were purchased from Integrated DNA Technologies (Coralville, IA). The pET-28a overexpression plasmid for *E. coli* was purchased from Novagen (MilliporeSigma, Burlington, MA). DNA sequencing was performed by Eurofins Genomics USA (Louisville, KY). Small molecule chemicals and bacterial media were purchased from Fisher Scientific (Waltham, MA), MilliporeSigma, AA Blocks (San Diego, CA), and CoALA Biosciences (Elgin, TX) and used without modifications. Enzymes and buffers for the molecular biology experiments were purchased from New England Biolabs (Ipswich, MA) and used as instructed. Mass spectrometry was performed with a Thermo Scientific TSQ Endura Mass Spectrometer in Dr. Guido F. Verbeck’s Laboratory for Imaging Mass Spectrometry (LIMS) in the Department of Chemistry and Biochemistry at Augusta University.

### Bioinformatics Analysis of HphA, IPMS, HCS, and hHphA

The amino acid sequence alignments between HphA (NCBI accession number: WP_012409019) and HCSs, as well as HphA and IPMSs, were performed in a previous study.^14^ The amino acid sequence of HphA from *Microcystis aeruginosa* NIES-4285 (hHphA) was obtained from the National Center for Biotechnology Information (NCBI; accession number: WP_130757515).^11^ The MultAlin tool (http://multalin.toulouse.inra.fr/multalin/) was used to align the amino acid sequences.^16^

The conservation level of five amino acid residues that were predicted to interact with the side chain of the substrate was analyzed as follows. The amino acid sequences for the homologues of HphA, HCS, and IPMS were obtained using the protein–protein Basic Local Alignment Search Tool (BLAST) for HphA and HCS, and Position-Specific Iterated BLAST for IPMS, limiting the search to 10,000 target sequences from the ClusteredNR database. The resulting hits were filtered using a threshold of 50% identity and 80% query coverage, meaning that all hits below these thresholds were excluded. This process identified 118 clusters for HphA, 92 clusters for HCS, and 9947 clusters for IPMS. Each set of sequences was aligned using Multiple Alignment using Fast Fourier Transform version 7 (MAFFT 7),^17,18^ and the conservation status was visualized using WebLogo 3 (Figure S1).^19,20^ All programs were run with their default settings, except for the number of target sequences in the BLAST search.

### Site-Directed Mutagenesis of HphA

Site-directed mutagenesis of HphA was conducted using overlap extension polymerase chain reaction (PCR).^21^ All PCRs were performed with Phusion DNA polymerase (New England Biolabs) using the following components: Phusion GC buffer, 0.2 mM dNTP, 20 pg/μL template, 0.5 mM forward and reverse primers, 3% dimethyl sulfoxide (DMSO), Phusion DNA polymerase, and water to a total volume of 50 μL. The PCR programs were optimized as follows: initial denaturation for 1 min at 95 °C, 30 cycles of 20 s at 95 °C, 30 s at 52 °C, and 2 min and 30 s at 72 °C, followed by a final extension for 5 min at 72 °C. The template for the site-directed mutagenesis was the HphA wild-type (WT) expression plasmid, pHphA-CHis-pET28a, which was constructed in a previous study.^14^ All HphA mutant expression plasmids were constructed so that the expressed protein contained a 6× His tag at the C-terminus. The mutagenesis began with synthesizing two DNA fragments that contained the corresponding mutations in their overlapped regions through PCR. This first round of PCR was performed using two primer pairs: (1) primer #1 with the reverse mutation primer (HphA-Mutation-R) and (2) primer #2 with the forward mutation primer (HphA-Mutation-F) (Table S1). These two PCR products were subsequently purified through agarose gel extraction (GeneJET, Thermo Scientific) and served as templates for the second round of PCR. In the second round of PCR, the primer pair consisting of primers #1 and #2 was employed to generate the full-length *hphA* mutant. This PCR product was also purified via agarose gel extraction and digested by the restriction enzymes NcoI and XhoI, along with the pET-28a plasmid. The digested *hphA* mutant and pET-28a were ligated with T4 DNA ligase, and the ligation product was then transformed into chemically competent *E. coli* DH5α. Each transformed single colony was cultured overnight to miniprep the recombinant plasmid, which was verified through double digestion followed by sequencing reactions.

### Overexpression and Purification of HphA Mutants

HphA mutants were overexpressed and purified for *in vitro* characterization. The methods for expression and purification of HphA mutants followed those used for HphA WT developed in a previous study.^14^ The recombinant expression plasmid was transformed into *E. coli* BL21­(DE3) cells. Three colonies were picked and cultured in 3 mL of LB medium containing 50 μg/mL kanamycin at 37 °C with shaking at 200 rpm until the culture reached the log phase and appeared cloudy. This culture was then inoculated into 1 L of LB medium with 50 μg/mL kanamycin and shaken under the same conditions until the optical density at 600 nm (OD_600_) reached approximately 0.5. After cooling the culture below 16 °C, 0.2 mM isopropyl 1-thio-β-d-galactopyranoside (IPTG) was added, and the culture was incubated overnight at 16 °C with shaking at 200 rpm. The cells were harvested by centrifugation at 4000 rpm for 15 min at 4 °C and were then resuspended in approximately 30 mL of the Ni-NTA binding buffer (25 mM Tris-HCl (pH 8.0), 400 mM NaCl, 5 mM imidazole, and 10% glycerol). Four cycles of sonication, each lasting 120 s with alternating 10 s “on” and 10 s “off,” were utilized to disrupt the cells. Cell debris was removed by centrifugation at 14,000 rpm for 45 min at 4 °C. The supernatant containing the solubilized proteins was incubated with Ni-NTA resin (Thermo Scientific) for 2 h at 4 °C with gentle rotation. The Ni-NTA resin was packed into a column and washed 10 times with 5 mL of the wash buffer (25 mM Tris-HCl (pH 8.0), 400 mM NaCl, 40 mM imidazole, 10% glycerol). The protein was eluted in three fractions, using 1 mL of the elution buffer (25 mM Tris-HCl (pH 8.0), 400 mM NaCl, 250 mM imidazole, and 10% glycerol) for each fraction. The first two elution fractions were dialyzed against a dialysis buffer (40 mM Tris-HCl (pH 8.0), 200 mM NaCl, 2 mM β-mercaptoethanol, and 10% glycerol). Three dialysis solutions were used, with each dialysis session lasting at least 3 h in a 1 L dialysis buffer. The protein concentration was measured using a spectrophotometer at λ = 280 nm. Finally, the protein solution was flash frozen in liquid nitrogen and stored at −80 °C.

### *In Vitro* Colorimetric Assays for HphA Mutants

Colorimetric assays for the HphA mutants were performed using Ellman’s reagent (5,5′-dithiobis­(2-nitrobenzoic acid) (DTNB))^15^ to measure product formation through the release of CoA following the enzymatic reaction. The reactions (100 μL) were prepared to a final concentration of 50 mM Tris-HCl (pH 7.5), 20 mM MgCl_2_, 20 mM KCl, 1 mM DTNB, 1.5 mM acetyl coenzyme A (AcCoA), 5 μM of enzyme, and 1 mM of substrate; these methods were previously developed for characterizing the wild-type HphA^11^ based on information gathered from testing the activity of IPMS.^22^ Substrates were added directly to the 96-well plate, and the rest of the reaction mixture was prepared separately and then added to the corresponding well to start the reaction, after which the plate was placed directly into the plate reader (xMark, Bio-Rad Laboratories, Hercules, CA). Reactions were conducted at room temperature (69 °F = 20.6 °C) and measured at 412 nm every 15 s with orbital mixing for 1 s in between measurements. After all the data were obtained, appropriate negative controls, detailed in each section, were subtracted from all points. All colorimetric assays were at least duplicated.

### Substrate Profile Establishment for HphA Mutants

Substrate profiling of HphA mutants was established using the same reaction and measurement conditions as those mentioned in *In Vitro* Colorimetric Assays for HphA Mutants section, with reactions run for 20 min each. To establish the activity of HphA single mutants, 3-methyl-2-oxobutanoic acid (MOBA) was used as a substrate for the IPMS mimics, and α-ketoglutaric acid (αKG) and oxaloacetic acid (OAA) were used for the HCS mimics, and homophenylpyruvic acid (hPPA) was used for the hHphA mimics. For all the A73 single mutants, the substrates were 4-hydroxyphenylpyruvic acid (4HPPA), MOBA, 4-methyl-2-oxovaleric acid (MOVA), αKG, OAA, hPPA, indole-3-pyruvic acid (InPA), 4-imidazolepyruvic acid (ImPA), and hydroxypyruvic acid (HPA). The ImPA substrate was in hydrobromide hydrate form (C_6_H_6_N_2_O_3_·*x*HBr·*y*H_2_O), so its weight was calculated assuming that *x* and *y* both equaled one, which meant that its formal weight was 253.05 g/mol. To measure the chemical hydrolysis of AcCoA in the reaction solution, a negative control was prepared by replacing the enzyme with dialysis buffer (Overexpression and Purification of HphA Mutants section) and the substrate with DMSO. The negative control solution was marginally simplified from our previous study on HphA WT, which incorporated the purified enzyme in the solution,^14^ due to the larger number of experiments conducted, the qualitative objective, and the minimal differences observed in the controls. For ImPA, which is known to exhibit significant absorbance with DTNB,^14^ an additional negative control was obtained using the reaction solution without the enzyme. The structures of the substrates used in this study and their expected products are listed in Figure 2. Since HphA A73G and A73D formed precipitate during the reaction, their reaction solutions were prepared with 10% glycerol. To prevent heat shock for these mutants, substrates were added to the microfuge tubes on ice to start the reactions and then immediately placed into the well plate for measurement. All reactions were duplicated. Percent activities of the mutants were calculated by averaging the absorbance differences from 0 to 20 min of each reaction. The activities were then converted to percentage form, with HphA WT with 4HPPA activity being set to 100%, and all other mutants were related to that number.

**Figure 2:**
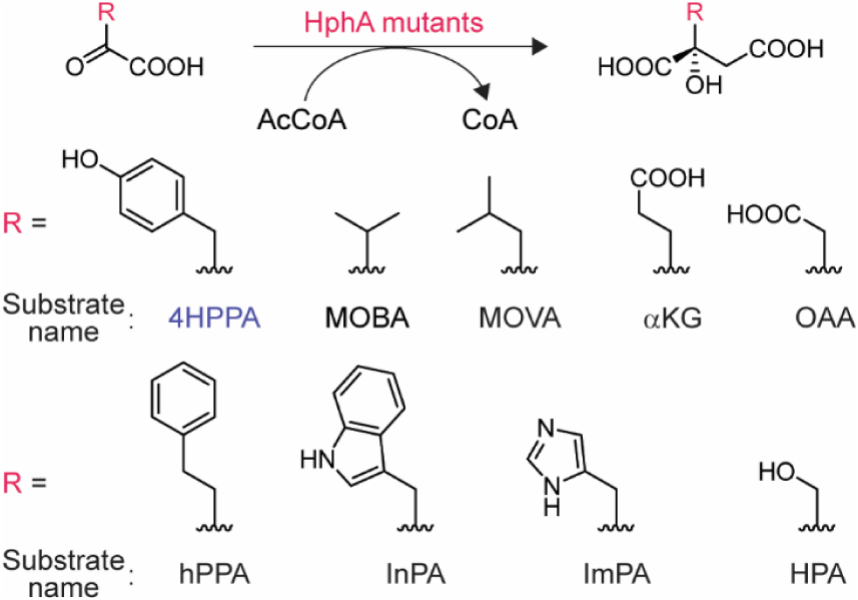
Structures of the substrates used in this study and their expected products. 4HPPA is one of the natural substrates of HphA WT and was used as a control.^14^ CoA = coenzyme A, AcCoA = acetyl-CoA, 4HPPA = 4-hydroxyphenylpyruvic acid, MOBA = 3-methyl-2-oxobutanoic acid, MOVA = 4-methyl-2-oxovaleric acid, αKG = α-ketoglutaric acid, OAA = oxalacetic acid, hPPA = homologated pyruvic acid (2-oxo-4-phenylbutanoic acid), InPA = indole-3-pyruvic acid, ImPA = 4-imidazolepyruvic acid, HPA = hydroxypyruvic acid.

### Kinetic Assays for HphA

The colorimetric assays to calculate the Michaelis–Menten kinetic parameters for each active mutant were established by using the same conditions as those previously mentioned (In *Vitro* Colorimetric Assays for HphA Mutants section). The kinetic assays were run for HphA A73L with 4HPPA, MOBA, MOVA, OAA, InPA, and HPA. The assays were also run for HphA WT with MOBA and MOVA, IPMS with MOBA and MOVA, and HCS with αKG and OAA. Substrate concentrations varied depending upon the enzyme and substrate pair, but these ranged from 20 mM to 0.002 mM. For HphA WT, MOBA concentrations were 0.05, 0.1, 0.2, 0.5, 1, 2, 4.2, 5, 8.4, and 20 mM and MOVA concentrations were 0.05, 0.2, 0.5, 1, 3, and 5 mM. For IPMS, MOBA concentrations were 0.02, 0.05, 0.2, 0.5, 1, and 3 mM, and MOVA concentrations were 0.002, 0.005, 0.02, 0.05, 0.2, and 0.5 mM. For HCS, αKG concentrations were 0.02, 0.05, 0.2, 0.5, 1, and 2 mM, and OAA concentrations were 0.2, 0.5, 1, and 5 mM. HCS formed a precipitate during the reaction, so the reactions were conducted with 10% glycerol and the substrate added on ice. For HphA A73L, 4HPPA concentrations were 0.01, 0.05, 0.1, 0.5, 1, and 3 mM; MOBA concentrations were 0.01, 0.05, 0.1, 0.5, 1, and 2 mM; MOVA concentrations were 0.01, 0.05, 0.1, 0.5, 1, 2, and 5 mM; OAA concentrations were 0.01, 0.05, 0.1, 0.5, and 1 mM; InPA concentrations were 0.005, 0.01, 0.05, 0.1, 0.5, 1, 2, and 3 mM; and HPA concentrations were 0.02, 0.05, 0.2, 0.5, 1, and 2 mM.

The data from colorimetric assays for each substrate were obtained by subtracting the negative control from the observed values. The negative control for kinetic assays was prepared similarly to that for the substrate profile assays (Substrate Profile Establishment for HphA Mutants section), with the following modifications made for greater accuracy. Instead of the dialysis buffer, the negative control consisted of a solution prepared using the same methods applied for protein stocks, as described in the Overexpression and Purification of HphA Mutants section, utilizing an empty pET-28a plasmid. For all substrates where the effects were negligible, 1 mM of each substrate was used for the negative control, except for InPA. The negative control for InPA was obtained for each concentration tested during the kinetics studies. The test enzyme was not included in the negative control because both substrates were present in this solution. The absorbance was converted to concentration utilizing a standard curve for thiol generated in-house using cysteine in the reaction solution of an identical volume. The calculated slope (*m* = ε × *l*; where ε is the extinction coefficient, and *l* is the path length) for absorbance (*A*) versus concentrations (*c*) was 3.866 mM^–1^. The concentrations of CoA generated by the reaction were calculated using the following equation:

$$c=\frac{A}{m}$$

The linear portions of the two reactions for each substrate concentration were taken and analyzed with GraphPad Prism (GraphPad Software, Boston, MA) to obtain Michaelis–Menten curves and parameters for each enzyme and substrate pair.

### Mass Spectrometry Analysis

Tandem mass spectrometry (MS/MS) with the negative mode of electron spray ionization (ESI) was performed to detect the product of the HphA A73L reaction performed with MOBA. Reactions were run overnight in the slightly modified reaction solution described in In *Vitro* Colorimetric Assays for HphA Mutants section, with 10 μM enzyme and 10 mM substrate. This reaction was quenched by the addition of 10 μL of 1 M HCl and then extracted four times with 100 μL of ethyl acetate, followed by rotary evaporation to remove the solvent. The dried sample was then prepared to be at a final concentration of 5 ppm in methanol with 1% NH_4_OH. The commercial standard of the expected product, 2-isopropylmalic acid (2-IPMA), was dissolved in methanol with 1% NH_4_OH for a final concentration of 5 ppm. Samples were run on a Product Ion Scan at the calculated parent mass of 175 [M – H]^−^ ± 1 Da with a collision energy of 15 V and analyzed with Xcalibur software (Thermo Scientific).

## Results and Discussion

### Bioinformatics to Design the Mutant That Mimics the Activity of Homologous Enzymes

To demonstrate the amenability of HphA to engineering, bioinformatics was used to design mutants of HphA that mimic the activity of homologous enzymes in primary metabolism. The enzymes homologous to HphA are HCS, IPMS (LeuA), and homoHphA (hHphA). HCS is the first enzyme involved in l-Lys biosynthesis after transamination, which is a regulated step that involves the condensation of AcCoA and αKG to form homocitrate and CoA.^23,24^ IPMS is the first enzyme involved in l-Leu biosynthesis, and it catalyzes the condensation of MOBA and AcCoA into 2-isopropylmalate and CoA.^25^ hHphA is hypothesized to be involved in the production of doubly (di)-homologated l-tyrosine (dil-hTyr).^11^

The bioinformatics analysis was performed in a previous study, which identified potential amino acid residues that are crucial for the selection of the substrate in HphA, HCSs, and IPMSs.^14^ It was first conducted with the most homologous structurally characterized enzyme, HCS from *Sulfolobus acidocaldarius* (PDB ID: 6KTQ, 28.9% sequence identity, 48.9% similarity to HphA),^24^ by taking advantage of this crystal structure binding the substrate αKG (Figure 3A). The amino acid sequence of another HCS from *Thermus thermophilus* (PDB ID: 2ZTJ, 28.9% identity, 45.5% similarity)^26^ was also aligned properly with HphA. These two HCSs were utilized to analyze the amino acid residues in the active site (Figure 3B). The amino acid sequence of HphA was also aligned with three structurally characterized IPMSs from *Neisseria meningitidis* serogroup B (PDB ID: 3RMJ, 28.7% identity, 44.9% similarity)^27^ from *Cytophaga hutchinsonii* (PDB ID: 3EEG, 26.2% identity, 40.5% similarity),^28^ and from *Listeria monocytogenes* serotype 4b str. F2365 (PDB ID: 3EWB, 28.5% identity, 44.6% similarity)^29^ (Figure 3C). These bioinformatic analyses identified five amino acid residues that may play a role in substrate selection, which were A73, D157, A159, M186, and S242 in HphA (Table 1).

**Figure 3:**
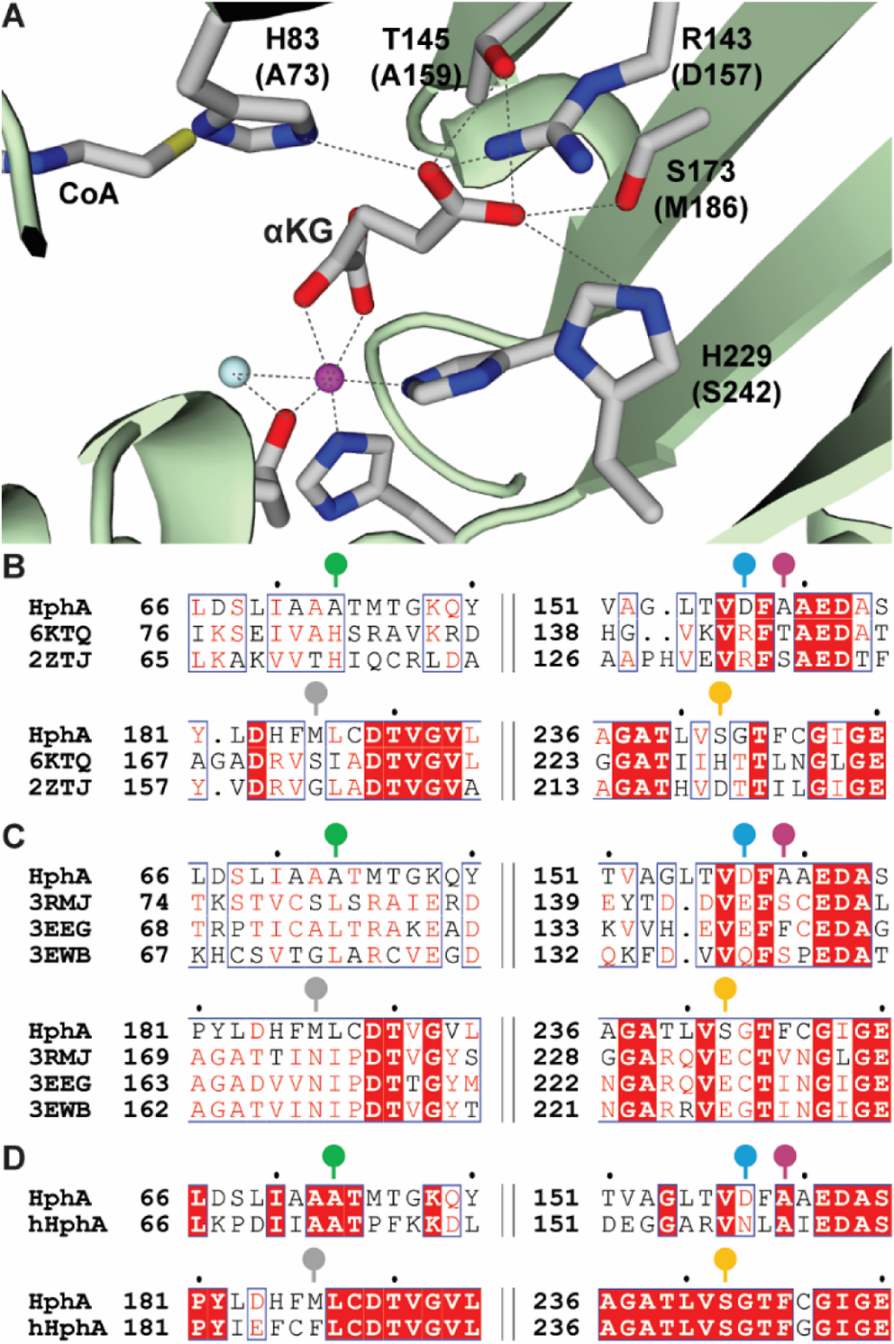
**A.** Active site of homocitrate synthase with αKG and CoA, focusing on interactions between the side chain of αKG and the amino acid residues of HCS (PDB ID: 6KTQ).^24^ The magenta and cyan balls represent Zn^2+^ and a water molecule, respectively. The amino acid residues in parentheses are those found in HphA. The dotted lines represent potential polar interactions. The amino acid sequence alignments are shown as follows: **B.** HphA, HCS from *S. acidocaldarius* (PDB ID: 6KTQ)^24^ and HCS from *T. thermophilus* (PDB ID: 2ZTJ).^26,27^ **C.** HphA, IPMS from *N. meningitidis* serogroup B (PDB ID: 3RMJ),^27^ IPMS from *C. hutchinsonii* (PDB: 3EEG),^28^ and IPMS from *L. monocytogenes* serotype 4b str. F2365 (PDB ID: 3EWB).^29^ **D.** HphA and hHphA from *M. aeruginosa* NIES-4285.^11^ The amino acid residues targeted in this study are represented by sticks with circular heads. The colors used correspond to those in Figure 4A-D. Panels B and C were adapted with permission from Stewart, L. E.; Owens, S. L.; Ahmed, S. R.; Lang Harman, R. M.; Mori, S., Characterization of HphA: The First Enzyme in the Homologation Pathway of l-Phenylalanine and l-Tyrosine. *ChemBioChem* 2024, 25 (16), e202400369. Copyright 2024 Wiley-VCH GmbH.

**Table 1:** Amino Acid Residues in the Active Site of HphA and Homologous Enzymes Hypothesized to Play Roles in the Substrate Selection^a^

Enzymes	HphA	HCS	IPMS	hHphA
**Amino acid residues**	Ala73	His	Leu	*Ala*
	Asp157	Arg	Glu	Asn
	Ala159	Thr	Ser	*Ala*
	Met186	Ser	Asn	Phe
	Ser242	His	Glu	*Ser*

a The amino acid residues that are identical in the homologous enzymes to those of HphA are italicized. The conserved residues within homologues of HphA, HCS, and IPMS are underlined. HCS = homocitrate synthase; IPMS = 2-isopropylmalate synthase; hHphA = homologous enzyme of HphA that was proposed to accept homologated 4-hydroxyphenylpyruvic acid as a substrate.

The conservation level of five amino acid residues that were predicted to be important for interacting with the side chain of the substrate was analyzed (Table 1 and Figure S1). All of these amino acid residues were found to be highly conserved in HCS and IPMS. This high conservation was anticipated, as these enzymes are involved in primary metabolism and take specific substrates into their respective homologation pathways. However, it was surprising to find that three of the five amino acid residues (A73, D157, and M186) in HphA were significantly less conserved. This lack of conservation may be attributed to the diversity of substrates accepted by the homologation pathway involving HphA. Anabaenopeptins form a large family of compounds with variations that include different combinations of homologated l-Phe and l-Tyr residues. These can range from containing only l-hPhe or l-hTyr to combinations of both, as well as variants with di- or tri-l-hTyr.^7^ These data suggest that residues A73, D157, and M186 may have more important roles than residues A159 and S242 in the selection of the substrate side chains, particularly for aromatic side chains.

In this study, the amino acid sequence of the recently discovered hHphA from *M. aeruginosa* NIES-4285, which has not been characterized functionally or structurally, was additionally aligned with HphA (Figure 3D and Figure S2).^11^ HphA and hHphA are highly homologous and display 71.7% sequence identity and 82.7% sequence similarity to each other. Based on this sequence alignment and the previous consensus of HCS and IPMS data with HphA, the D157 and M186 residues were not conserved in HphA and hHphA and were therefore predicted to be the key residues for expanding the substrate promiscuity to a slightly larger side chain. Based on the bioinformatics data, we designed mutations to mimic the activity of HCS, IPMS, or hHphA; specific amino acid mutations are shown in Table 1.

### Substrate Alteration of Single Mutants That Mimic the Homologous Enzymes

The catalytic functions of the single mutants of HphA, which mimic the active site of the homologous enzymes HCS and IPMS, were evaluated through *in vitro* enzymatic assays. Their relative activities were measured through colorimetric assays, using the substrates αKG and OAA for the HCS mimic, as well as MOBA for the IPMS mimic. Since the reactions catalyzed by HphA and its homologous enzymes produce CoA as a coproduct, Ellman’s reagent was used to detect the free thiol.^15^ Activity was determined after the reactions had proceeded for 20 min; each reactivity was related to HphA WT with the same substrate, which was set to 100% activity (Figure 4A-D).

**Figure 4:**
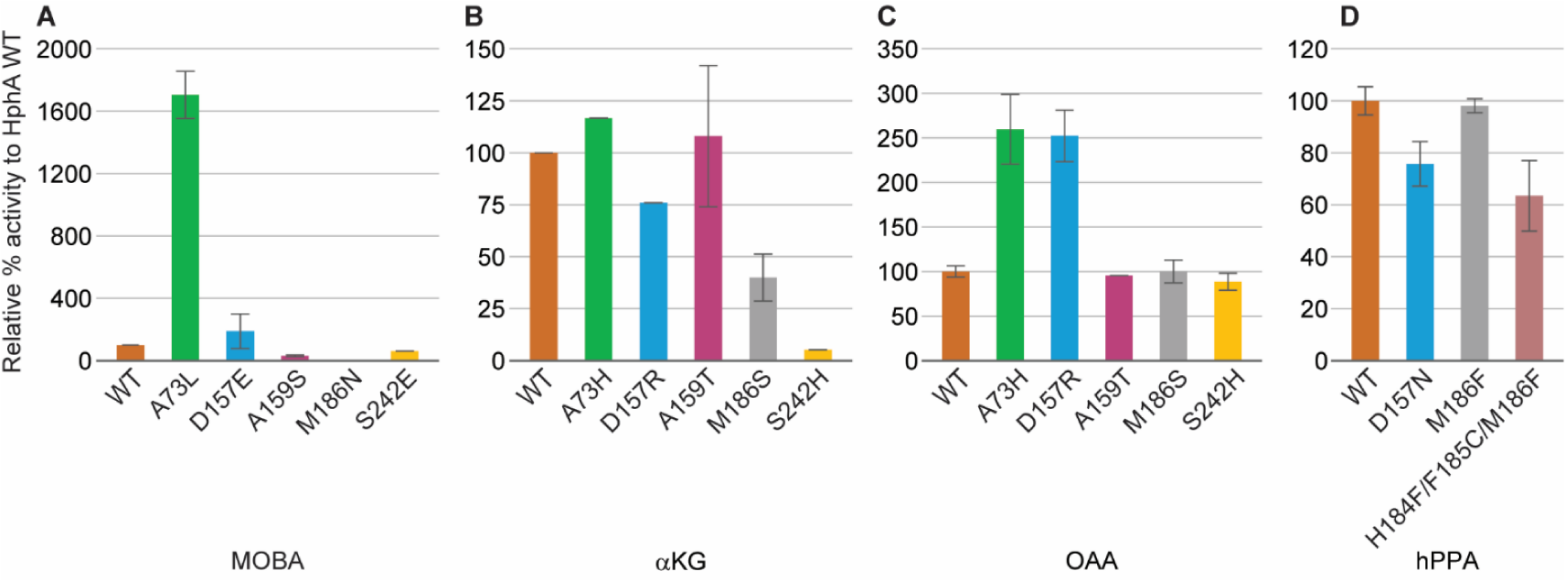
Relative % activity of HphA mutants to HphA WT for mimicking: **A.** the IPMS active site with MOBA, **B.** the HCS active site with αKG, **C.** the HCS active site with OAA, and **D.** the hHphA active site with hPPA. MOBA = 3-methyl-2-oxobutanoic acid, αKG = α-ketoglutaric acid, OAA = oxaloacetic acid, hPPA = homologated pyruvic acid (2-oxo-4-phenylbutanoic acid). The colors used correspond to those in Figure 3B-D. The error bars represent the range of the data (*n* = 2).

HphA single mutants that mimic the IPMS active site were A73L, D157E, A159S, M186N, and S242E. The data revealed that HphA A73L enhanced the activity with MOBA 17-fold compared to the WT. HphA D157E displayed slightly higher activity than the WT (1.9-fold), while the other IPMS mimics showed less activity than the WT (Figure 4A).

HphA single mutants that mimicked the HCS active site included A73H, D157R, A159T, M186S, and S242H. These mutants were tested with αKG, but none of the mutants showed enhanced activity with the substrate compared to that of the WT (Figure 4B). Consequently, we tested the activity of these mutants with an analogous compound, namely OAA (Figure 4C). OAA is the substrate of citrate synthase (CS), which is another homologous enzyme to HphA and is involved in the citric acid cycle in eukaryotes.^30^ OAA was approximately 2.5-fold better as a substrate for both HphA A73H and D157R compared to the WT, while the other mutants did not display enhanced activity with this compound.

The data obtained from the single mutants designed to mimic the active sites of IPMS and HCS suggest that the A73 residue plays a role in substrate selectivity. These results were unexpected based on the bioinformatic analysis of the active site of hHphA, as discussed in the previous section Bioinformatics to Design the Mutant That Mimics the Activity of Homologous Enzymes, which predicted the importance of residues D157 and M186 in the substrate selectivity of HphA. This initial observation was confirmed by evaluating the activity of HphA mutants that mimic the hHphA active site with hPPA, including the single mutants, D157N and M186F, and the triple mutant, H184F/F185C/M186F, whose residues form a portion of the β-sheet in the active site. None of these mutants showed increased activity with hPPA (Figure 4D).

One of the IPMS mimics, A73L, displayed a significantly greater increase in activity with its substrate compared with the HCS mimic, A73H. This difference can be attributed to the distinct nature of the substrate side chains. Specifically, the types of substrates for each enzyme are as follows: HphA interacts with nonpolar or slightly polar aromatic groups (Phe and Tyr), IPMS with a nonpolar aliphatic group (Val), and HCS with a polar acidic group (Glu). Therefore, it is reasonable to conclude that mimicking the IPMS active site is simpler than mimicking the HCS active site. As a result, we expanded the set of HphA mutants designed to mimic the HCS active site by introducing additional mutations.

### Expansion of the Mutations for Polar Substrates

To enhance the HCS mimic activity with αKG and OAA, we created double mutants of HphA based on the A73H mutation. We incorporated A73H into each of the double mutants due to its apparent impact on substrate selectivity and its demonstrated higher activity with OAA than HphA WT. The other mutation in each double mutant was selected from the previously tested single mutants. We particularly anticipated positive outcomes from the A73H/D157R combination while still considering A159T, M186S, and S242H as potentially important residues in the active site.

The results of activity tests of the double mutants, compared to HphA WT and A73H, are shown in Figure 5. Unfortunately, none of the double mutants had significantly more activity against OAA and/or αKG than the single A73H mutant; even the A73H/D157R double mutant did not have higher activity, contradicting expectations. It is important to note that all double mutations caused the enzyme to be less stable: reaction solutions began to form a precipitate after 30 min of the reaction at room temperature.

**Figure 5:**
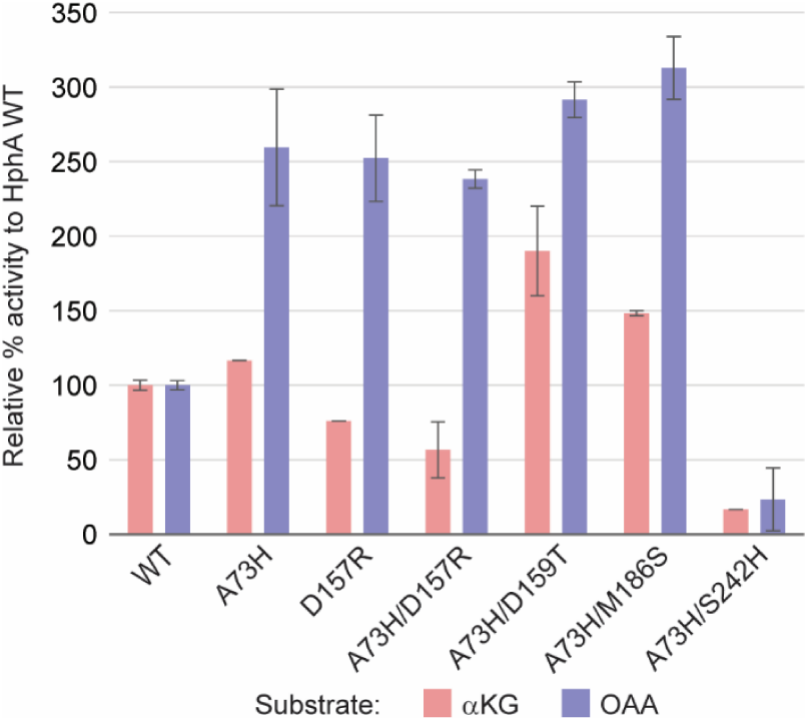
Relative % activity to HphA WT of HphA double mutants that mimic the HCS active site with αKG (pink) and OAA (blue) as substrates. The error bars represent the range of the data (*n* = 2).

### Expansion of the A73 Mutations for Various Substrates

Following the evaluation of the single mutants designed to mimic the active sites of IPMS and HCS, the A73 residue was studied further to create mutants that accept a wide range of substrates. Other than A73L and A73H, we designed six more mutations from A73 and tested their activities compared to HphA WT on nine different substrates.

Based on the data obtained from HphA A73L and A73H, we selected to mutate the A73 residue to V, G, F, N, D, and S. The A73V mutant was designed to slightly expand the active site binding pocket from the A73L mutant, anticipating that MOBA and/or MOVA (containing the Leu side chain) could act as substrates. The A73G mutant was developed to increase activity with compounds larger than the natural substrates of HphA WT, phenylpyruvic acid (PPA) and 4HPPA, such as hPPA. Conversely, the A73F mutant was aimed at making the active site smaller to see if the substrate scope was modified accordingly. The mutations to increase the polarity, which were A73N, A73D, and A73S, were intended to create higher flexibility in the active site than the A73H mutation for substrates with polar side chains.

Each mutation was tested with nine different commercially available substrates (Figures 2 and 6). 4HPPA is one of the natural substrates of HphA and was used as a control. MOBA, αKG, and OAA are the natural substrates of IPMS, HCS, and CS, respectively, as discussed in previous sections. MOVA is the homologated version of MOBA and was included to evaluate if the active site with a smaller binding pocket better utilizes this compound. hPPA was used to test if dihomologation can be initiated by a mutant HphA. HPA is the Ser counterpart of PPA and 4HPPA. All of the above homologated compounds can be found in nature. InPA and ImPA are the Trp and His counterparts of PPA and 4HPPA, respectively, in which homologated versions (hTrp and hHis) have not been found in nature.

**Figure 6:**
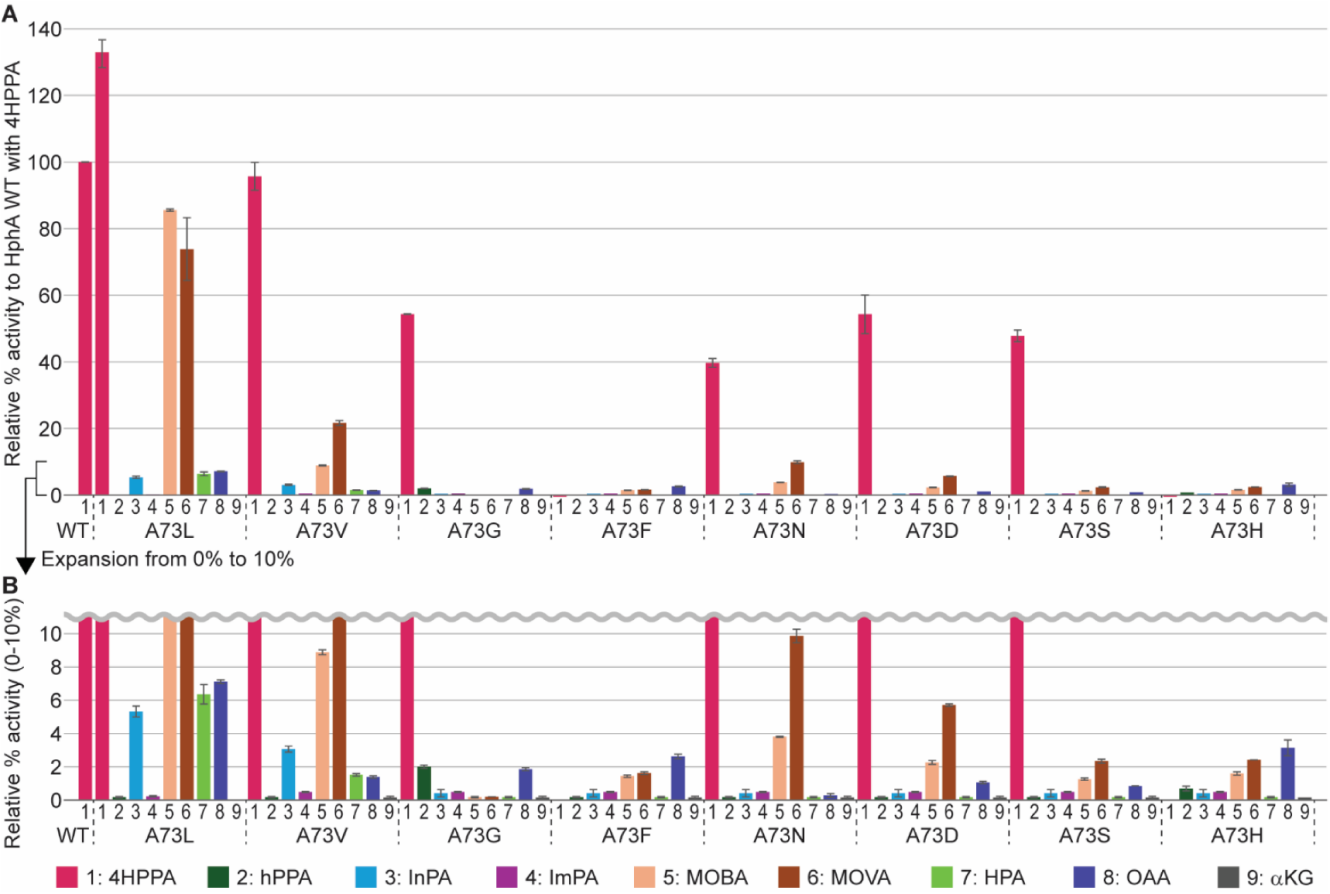
**A.** Relative % activity of A73 series mutant with various substrates to HphA WT with the natural substrate 4HPPA. **B.** The expansion of the *y*-axis of panel A from 0% to 10%. The error bars represent the range of the data (*n* = 2).

The activity of the mutants with each substrate was compared with the activity of HphA WT with 4HPPA (Figure 6). Contrary to our expectations, HphA A73L, which was intended to mimic the IPMS active site, was the most active of all the mutants across all substrates, except for hPPA, ImPA, and αKG. The A73L mutant showed activity with 4HPPA (133%), InPA (5.3%), and MOBA (86%; as also presented in Figure 4A), MOVA (74%), HPA (6.4%), and OAA (7.1%). All mutations showed negligible activity with ImPA or αKG, while hPPA was only slightly active with the A73G mutant (2.0%). The polar substrates, ImPA, HPA, OAA, and αKG, were anticipated to be more active with the polar mutations (A73N, A73D, A73S, and A73H), which was not supported by the data. Mutations to large amino acid side chains, A73F and A73H, almost completely disrupted the HphA activity, including that with 4HPPA. These results may not be only due to the disruption of the substrate binding; since the A73 residue is also close to the CoA binding site (Figure 3A), mutations to the large side chains may damage the binding of CoA in the active site.

### Kinetics of HphA A73L Compared to Homologous Enzymes

To better understand the reactions catalyzed by HphA A73L, the Michaelis–Menten kinetic parameters were determined with active substrates, including 4HPPA, InPA, MOBA, MOVA, HPA, and OAA. Michaelis–Menten parameters were also determined for HphA WT with MOBA and MOVA, IPMS with MOBA and MOVA, and HCS with αKG and OAA (Table 2 and Figures S3 and S4). Kinetic data for HphA WT with 4HPPA were reported previously, with a *k*_cat_/*K*_m_ of 3.4 ± 0.4 mM^–1^ s^–1^.^14^ All kinetic assays were performed using the same methods as those for HphA with 4HPPA, except slight modifications were made for HCS. Due to the instability of HCS under the assay conditions, its stability was enhanced by adding 10% glycerol and starting the reactions on ice. As a result, comparisons of the activity between HphA A73L and HCS were not very reliable.

**Table 2:** Kinetic Data for HphA WT, Homologous Enzymes, and HphA A73L Mutant^a^

Enzyme	Substrate	*k*_cat_ (s^–1^)	*K*_m_ (mM)	*k*_cat_/*K*_m_ (mM^–1^ s^–1^)
**HphA WT**	4HPPA^14^	0.14 ± 0.01	0.040 ± 0.004	3.4 ± 0.4
	MOBA	0.0078 ± 0.0008	4.0 ± 1.1	0.0019 ± 0.0006
	MOVA	ND	ND	*0.0034 ± 0.0001
**IPMS**	MOBA	0.70 ± 0.05	0.11 ± 0.04	6.4 ± 2.1
	MOVA	0.013 ± 0.001	0.011 ± 0.002	1.1 ± 0.2
**HCS**	OAA	ND	ND	*0.0060 ± 0.0003
	αKG	0.065 ± 0.005	0.92 ± 0.15	0.070 ± 0.013
**HphA A73L**	4HPPA	0.22 ± 0.01	0.41 ± 0.05	0.54 ± 0.08
	MOBA	0.12 ± 0.01	0.65 ± 0.15	0.18 ± 0.05
	MOVA	0.10 ± 0.01	1.6 ± 0.4	0.064 ± 0.015
	OAA	0.0069 ± 0.0002	ND	ND
	HPA	0.0046 ± 0.0007	3.2 ± 1.0	0.0014 ± 0.0005
	InPA	0.0091 ± 0.0004	0.21 ± 0.04	0.043 ± 0.007

a 4HPPA = 4-hydroxyphenylpyruvic acid; MOBA = 3-methyl-2-oxobutanoic acid; MOVA = 4-methyl-2-oxovaleric acid; OAA = oxaloacetic acid; HPA = hydroxypyruvic acid; InPA = indole-3-pyruvic acid; αKG = α-ketoglutarate. ND represents nondeterminable data due to unmeasurable *K*_m_ values. The *k*_cat_/*K*_m_ values marked with an asterisk are estimated values based on the slope of the linear portion of the Michaelis–Menten plot. The data for HphA WT with 4HPPA were obtained in a previous study.^14^

The kinetics of HphA A73L with 4HPPA showed a *k*_cat_/*K*_m_ of 0.54 ± 0.08 mM^–1^ s^–1^, which was 6.3-fold lower than that of the HphA WT. This result was anticipated because the A73L mutation was designed to accept IPMS substrates. The kinetics for IPMS with MOBA and MOVA showed *k*_cat_/*K*_m_ values of 6.4 ± 2.1 mM^–1^ s^–1^ and 1.1 ± 0.2 mM^–1^ s^–1^, respectively. IPMS was very efficient with MOBA, its natural substrate, as well as decently efficient with MOVA, the homologated version of MOBA. As compared to these values, HphA WT displayed *k*_cat_/*K*_m_ values that were 3300-fold and 240-fold lower for MOBA and MOVA, respectively. The poor activity of HphA WT with MOBA and MOVA was previously demonstrated qualitatively, as MOBA showed only a few % activity compared to 4HPPA after a 20-min reaction.^14^ The *k*_cat_/*K*_m_ values for HphA A73L with MOBA and MOVA were anticipated to be greater than those of HphA WT due to its mutation to be an IPMS mimic. Its *k*_cat_/*K*_m_ value for MOBA was 0.18 ± 0.05 mM^–1^ s^–1^, which was 35-fold lower and 95-fold higher than those of IPMS and HphA WT, respectively. Similarly, the HphA A73L kinetic value for MOVA was 0.064 ± 0.015 mM^–1^ s^–1^, which was 17-fold lower and 14-fold higher than those of IPMS and HphA WT, respectively. These values showed that the A73L mutation on HphA significantly expanded the substrate scope for MOBA and MOVA, but it still did not reach the activity level of IPMS.

The kinetics for HphA A73L were also investigated using other low-activity substrates: OAA, HPA, and InPA. Although all of these substrates exhibited much lower *k*_cat_ values compared to 4HPPA, MOBA, and MOVA, their *K*_m_ values suggested unique interaction modes between HphA A73L and these substrates. The *K*_m_ value for OAA was notably low, rendering it unmeasurable with our colorimetric assays, and this value was the lowest among all combinations in this study. Given the low production of CoA in the substrate profile experiments involving HphA A73L and OAA, the product may not be efficiently released from the enzyme’s active site. This inhibits the substrate from accessing the mutant’s active site, resulting in a low *k*_cat_ value of 0.0069 ± 0.0002 s^–1^.

Among the measurable substrates, HphA A73L showed the lowest *k*_cat_/*K*_m_ value with HPA, calculated at 0.0014 ± 0.0005 mM^–1^ s^–1^. This low value was attributed to its high *K*_m_ value of 3.2 ± 1.0 mM, indicating weak interactions between the mutant enzyme and the substrate. Although the A73L mutation made the active site more accessible for HPA, the polar side chain may have adversely affected substrate binding.

In contrast, HphA A73L demonstrated a relatively good *k*_cat_/*K*_m_ value of 0.043 ± 0.007 mM^–1^ s^–1^ with InPA, attributable to its lower *K*_m_ value of 0.21 ± 0.04 mM compared to other substrates. The *k*_cat_/*K*_m_ value with InPA was 8.0%, 24%, and 67% of the corresponding values for 4HPPA, MOBA, and MOVA, respectively. This outcome suggests that since the natural substrates of HphA WT have aromatic side chains, the mutant that becomes less specific retains the ability to interact with aromatic side chains, even though the indole group differs significantly in size and polarity from phenyl and phenol groups. These measurable activities with different substrates demonstrated that HphA A73L has a wider range of substrate scopes than we anticipated from the bioinformatic study, showing that HphA is amenable to engineering in its active site.

### Generation of the Noncanonical Product by HphA A73L

To confirm that the reactions catalyzed by HphA A73L were producing the anticipated product from the noncanonical substrate, MOBA, tandem mass spectrometry (MS/MS) analysis was carried out. We performed the reaction of HphA A73L with MOBA under conditions slightly modified from those used in colorimetric assays. Mass spectrometry was then conducted on the extracted product, focusing on obtaining the [M – H]^−^ peak and interpreting the fragmentation pattern. This was compared to the mass spectrometry data taken with the commercial standard 2-IPMA (Figure 7). Key fragment peaks observed in the standard sample were also present in the reaction product sample. These results showed that the expected product was successfully produced from the reaction catalyzed by HphA A73L, demonstrating the functionality of the mutant with a noncanonical substrate.

**Figure 7:**
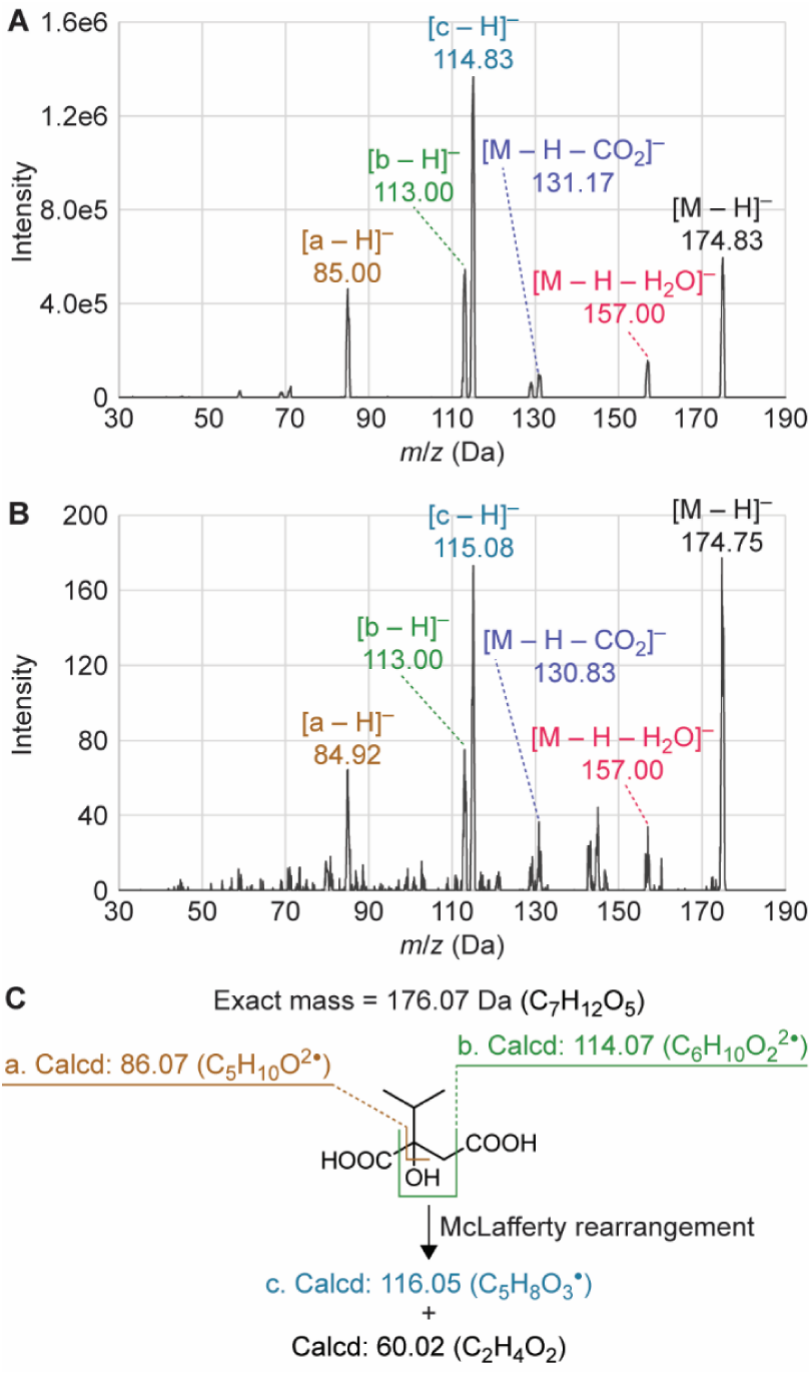
MS/MS analysis for the product of the reaction catalyzed by HphA A73L with the substrate MOBA. **A.** The commercially available product, 2-IPMA. **B.** Extracted sample from the HphA A73L reaction with MOBA. **C.** Possible fragmentation patterns on 2-IPMA. The molecular formulas with labels “a”-“c” correspond to the peaks on each MS/MS chromatogram.

## Conclusion

HphA, which is part of the homologation pathway in secondary metabolites of cyanobacteria, has previously been shown to be a substrate-specific enzyme and acts as a gatekeeper of the pathway. The homologation pathway, once characterized and engineered, has the potential to be used in the derivatization of bioactive peptide molecules; therefore, the goal of this project was to demonstrate the amenability of HphA to engineering for future research. Through bioinformatic studies using the homologous enzymes HCS, IPMS, and hHphA, several potential amino acid residues in the active sites were revealed for the alteration of the substrate scope; these locations were targeted for mutations of HphA. Time-course assays with the mutants, mimicking the active sites of HCS and IPMS, identified the A73 residue as being key in the substrate specificity of HphA. Subsequently, a total of eight mutations on the A73 residue were conducted to expand or alter the substrate scope. The A73L mutant demonstrated activity with a much wider range of substrates than the WT; these substrates included amino acid counterparts of Val, Leu, Ser, Trp, and Asp, in addition to the natural substrate of the WT, Tyr, out of nine substrates tested. Significantly, activity of this mutant with the Val counterpart, MOBA, increased 95-fold from that of the WT. Moreover, acceptance of the Trp counterpart, InPA, as a substrate was intriguing, as the homologated Trp (hTrp) does not occur in nature, and the synthetic compound is not readily commercially available. Optimizing the substrate scope for Trp would lead to the production of hTrp by the homologation pathway, which will provide an additional scaffold for drug synthesis. As only one mutation in the bioinformatically analyzed active site significantly relaxed the substrate specificity, it could be optimized further for specific noncanonical substrates or for higher promiscuity by other methods, such as structure-guided mutagenesis, following X-ray crystallography, and directed evolution. This initial engineering study to prove the amenability of HphA promises hopeful applications in the homologation pathway.

## Supplementary Material



## Notes

Includes visualized conservation level of amino acid residues in the active sites of HphA, IPMS, and HCS (Figure S1); full amino acid sequence alignment between HphA and hHphA (Figure S2); Michaelis–Menten kinetic plots of HphA A73L (Figure S3); Michaelis–Menten kinetic plots of HphA WT, IPMS, and HCS (Figure S4); and PCR primers used in this study (Table S1) (PDF)
