# Supplementary material for: Amenability of the Gatekeeper Enzyme HphA to Engineering in the Homologation Pathway of l‑Phenylalanine and l‑Tyrosine through Homology-Based Site-Directed Mutagenesis
Source: ACS Omega. 2026 Feb 17;11(8):13789–98. doi: 10.1021/acsomega.5c12112 (PMC12961565; doi:10.1021/acsomega.5c12112)
Supplement: Supplementary file 1 [file ao5c12112_si_001.pdf]

Amenability of the Gatekeeper Enzyme HphA to Engineering in the Homologation Pathway of L-Phenylalanine and L-Tyrosine through Homology-Based Site-Directed Mutagenesis

Rebecca M. Lang Harman,[1] H. Grace Blackstone,[1] Favor O. Aruna,[2] Shivam R. Patel,[2] Minho Shin,[2] Reed K. NeSmith,[2] D. Brooks Dickson,[2] Angela C. Spencer,[1] and Shogo Mori[1*]

[1]Department of Chemistry and Biochemistry, College of Science and Mathematics, Augusta University, Augusta, Georgia 30912, USA

[2]Department of Biological Sciences, College of Science and Mathematics, Augusta University, Augusta, Georgia 30912, USA

Outline:

Figures and Tables

Figure S1. Visualized conservation level of active site amino acid residues 3

Figure S2. Amino acid sequence alignment between HphA and hHphA 4

Figure S3. Kinetic assays 5

Figure S4. Kinetic assays 2 6

Table S1. PCR primers used in this study 7

References 8

Figures and Tables

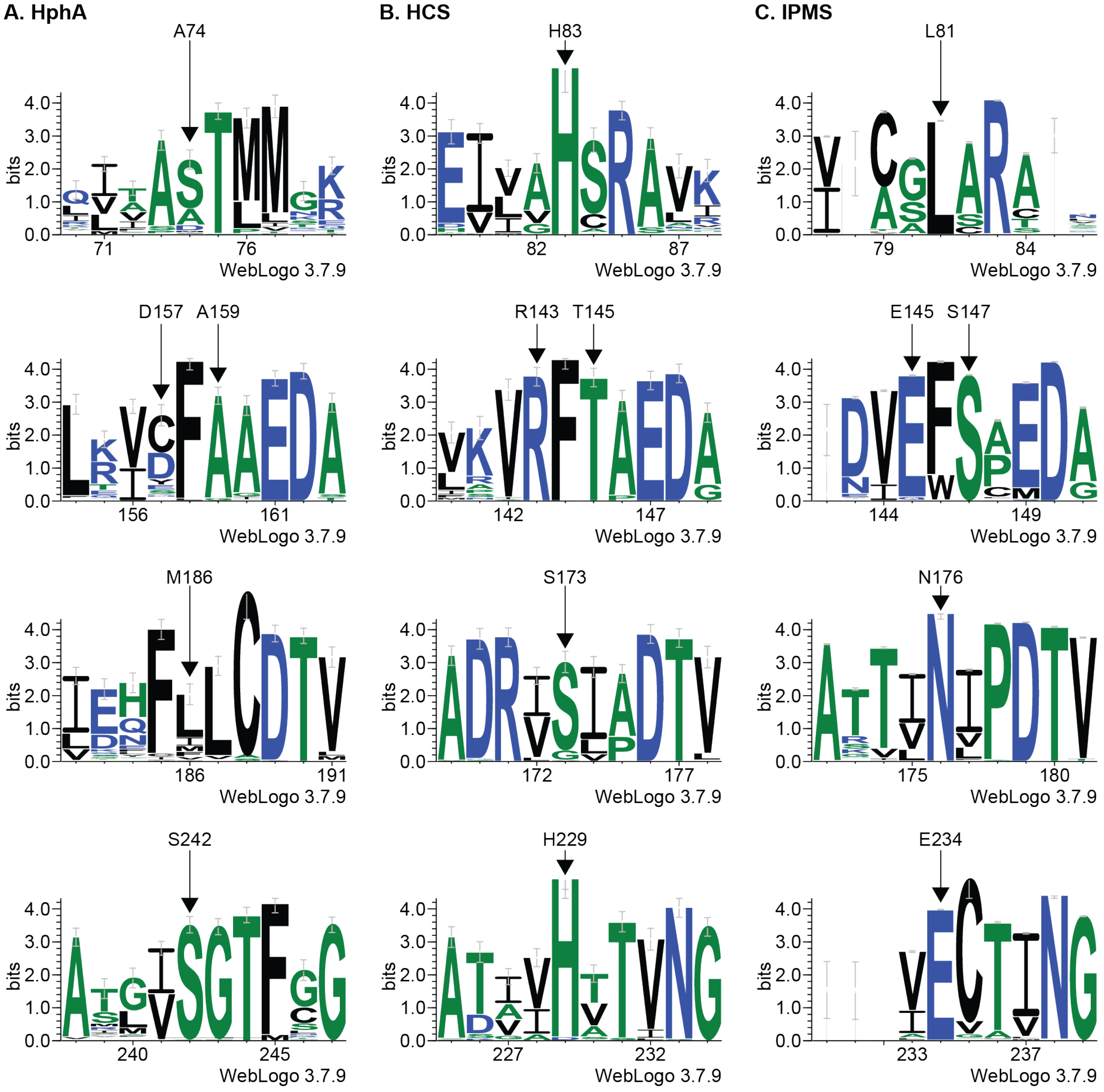

Figure S1. Conservation level of active site amino acid residues for **A.** HphA, **B.** homocitrate synthase (HCS), and **C.** 2-isopropylmalate synthase (IPMS), visualized by WebLogo 3. Each x-axis represents the amino acid residue number for HphA (UniProt B2J8P8),[1] HCS (PDB 6KTQ),[2] and IPMS (PDB 3RMJ),[3] used for amino acid sequence alignment (Figures 3B and 3C).

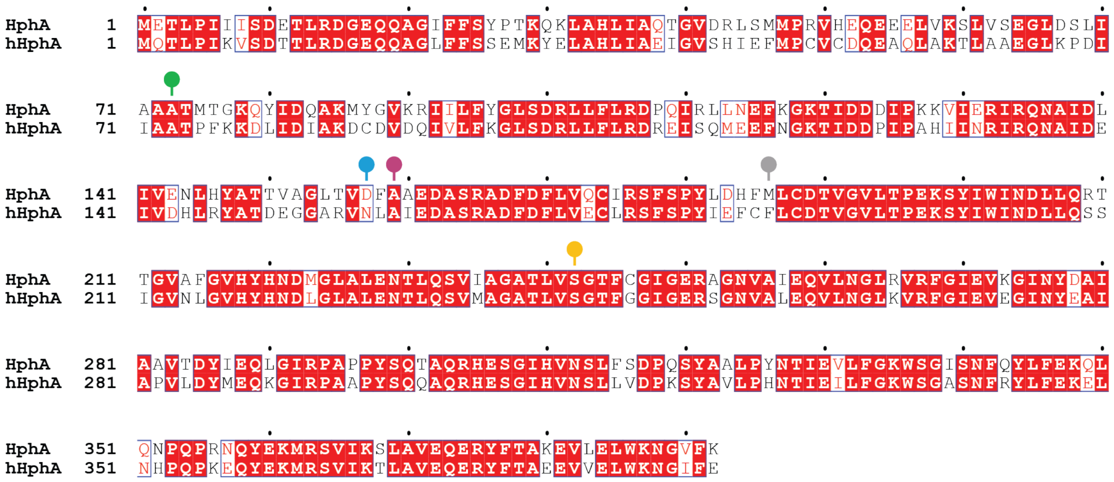

Figure S2. The full amino acid sequencing alignment between HphA and hHphA.[4, 5] Each pin represents the potential amino acid residue in the active site, which plays a role in the substrate selectivity.

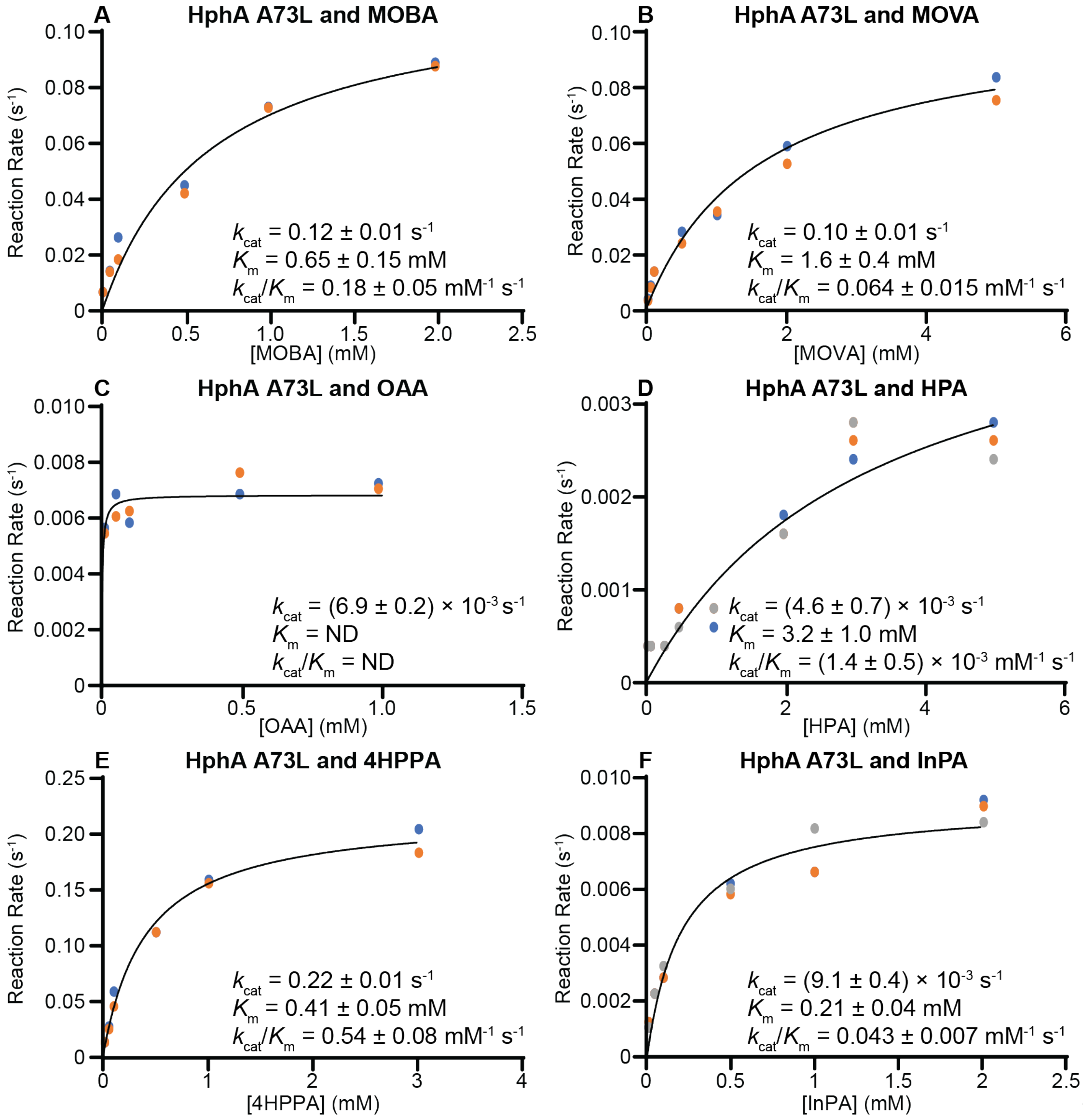

the

Figure S3. Michaelis–Menten kinetics of HphA A73L with various substrates. **A.** HphA A73L with 3-methyl-2-oxobutanoic acid (MOBA); **B.** HphA A73L with 4-methyl-2-oxovaleric acid (MOVA); **C.** HphA A73L with oxaloacetic acid (OAA); **D.** HphA A73L with hydroxypyruvic acid (HPA); **E.** HphA A73L with 4-hydroxyphenylpyruvic acid (4HPPA); **F.** HphA A73L with indole-3-pyruvic acid (InPA). ND represents non-determinable data due to unmeasurable K_m values. All assays were at least duplicated, and the data was individually plotted.

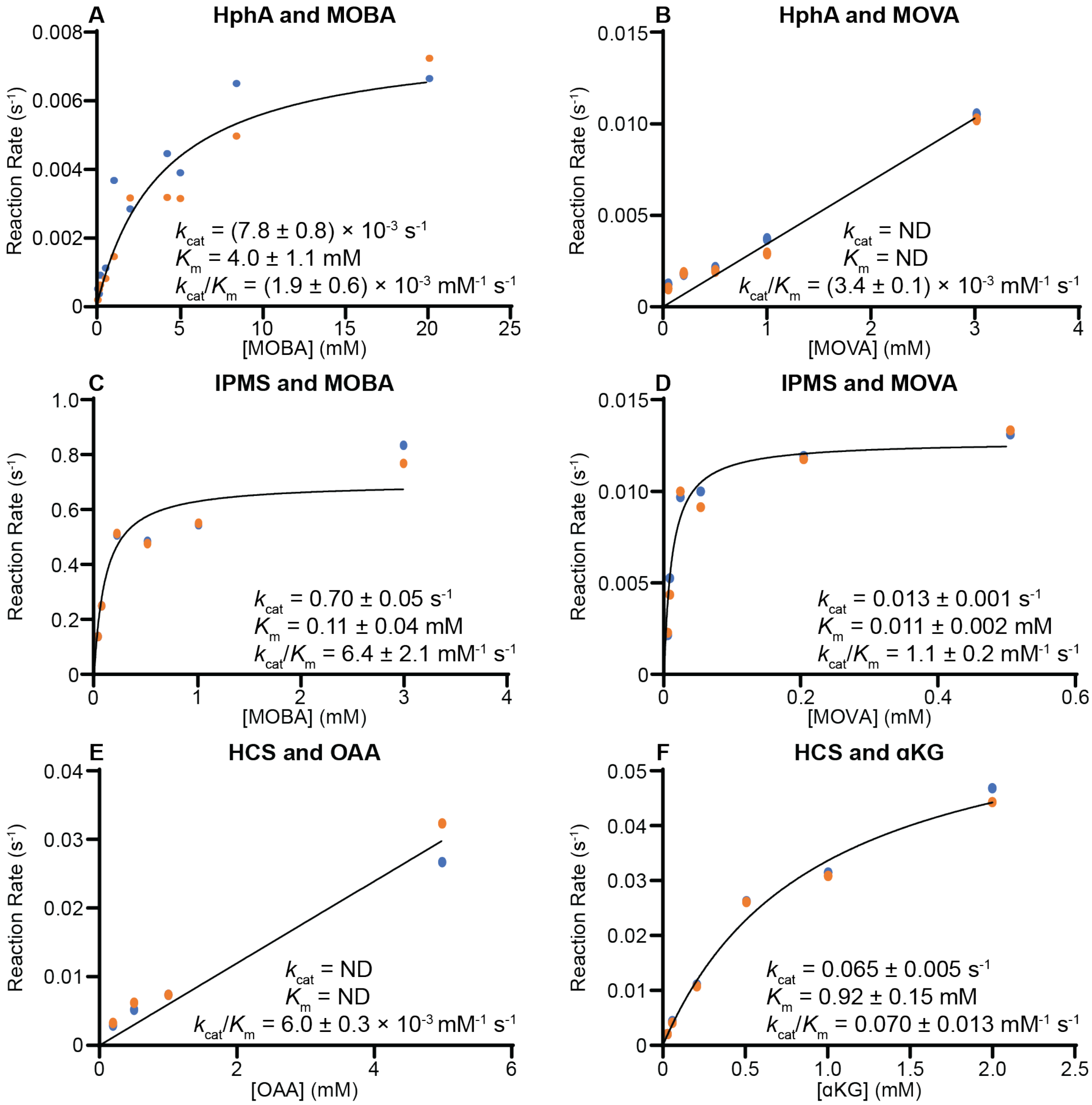

Figure S4. Michaelis–Menten Kinetics of HphA and homologous enzymes. **A.** HphA and 3-methyl-2-oxobutanoic acid (MOBA); **B.** HphA and 4-methyl-2-oxovaleric acid (MOVA); **C.** isopropylmalate synthase (IPMS) with MOBA; **D.** IPMS with MOVA; **E.** homocitrate synthase (HCS) with oxaloacetic acid (OAA); **F.** HCS with α-ketoglutaric acid (αKG). The k_{cat}/K_m values for HphA with MOVA as well as HCS with OAA were estimated from the slope of the initial portion of the Michaelis-Menten plot. ND represents non-determinable data due to unmeasurable K_m values. All assays were duplicated, and the data was individually plotted.

Table S1. PCR primers used in this study

Primer #	Primer name	Primer sequence	5' or 3'
1	HphA-NcoI-F	CTAAAA<u>CCATGG</u>AAACCCTTCCTATCATA	5'
2	HphA-XhoI-R-noSTOP	GAAGAT<u>CTCGAG</u>TTTAAACACCCCATTTTTC	3'
3	HphA-A73L-F	CTGATCGCTGCT***CTT***ACCATGACGGGA	5'
4	HphA-A73L-R	TCCCGTCATGGT***AAG***AGCAGCGATCAG	3'
5	HphA-D157E-F	GGACTCACTGTA***GAG***TTTGCCGCCGAAG	5'
6	HphA-D157E-R	CTTCGGCGGCAAA***CTC***TACAGTGAGTCC	3'
7	HphA-A159S-F	CACTGTAGACTTT***TCC***GCCGAAGATGCC	5'
8	HphA-A159S-R	GGCATCTTCGGC***GGA***AAAGTCTACAGTG	3'
9	HphA-M186N-F	CTCGACCATTTT***AAC***CTCTGTGATACC	5'
10	HphA-M186N-R	GGTATCACAGAG***GTT***AAAATGGTCGAG	3'
11	HphA-S242E-F	GCGACTTTAGTA***GAG***GGTACGTTTTGC	5'
12	HphA-S242E-R	GCAAAACGTACC***CTC***TACTAAAGTCGC	3'
13	HphA-A73H-F	CTGATCGCTGCT***CAT***ACCATGACGGGA	5'
14	HphA-A73H-R	TCCCGTCATGGT***ATG***AGCAGCGATCAG	3'
15	HphA-D157R-F	GGACTCACTGTA***CGC***TTTGCCGCCGAAG	5'
16	HphA-D157R-R	CTTCGGCGGCAAA***GCG***TACAGTGAGTCC	3'
17	HphA-A159T-F	CACTGTAGACTTT***ACC***GCCGAAGATGCC	5'
18	HphA-A159T-R	GGCATCTTCGGC***GGT***AAAGTCTACAGTG	3'
19	HphA-M186S-F	CTCGACCATTTT***AGC***CTCTGTGATACC	5'
20	HphA-M186S-R	GGTATCACAGAG***GCT***AAAATGGTCGAG	3'
21	HphA-S242H-F	GCGACTTTAGTA***CAC***GGTACGTTTTGC	5'
22	HphA-S242H-R	GCAAAACGTACC***GTG***TACTAAAGTCGC	3'
23	HphA-A73G-F	CTGATCGCTGCT***GGT***ACCATGACGGGA	5'
24	HphA-A73G-R	TCCCGTCATGGT***ACC***AGCAGCGATCAG	3'
25	HphA-A73V-F	CTGATCGCTGCT***GTT***ACCATGACGGGA	5'
26	HphA-A73V-R	TCCCGTCATGGT***AAC***AGCAGCGATCAG	3'
27	HphA-A73F-F	CTGATCGCTGCT***TTT***ACCATGACGGGA	5'
28	HphA-A73F-R	TCCCGTCATGGT***AAA***AGCAGCGATCAG	3'
29	HphA-A73S-F	CTGATCGCTGCT***TCT***ACCATGACGGGA	5'
30	HphA-A73S-R	TCCCGTCATGGT***AGA***AGCAGCGATCAG	3'
31	HphA-A73D-F	CTGATCGCTGCT***GAT***ACCATGACGGGA	5'
32	HphA-A73D-R	TCCCGTCATGGT***ATC***AGCAGCGATCAG	3'
33	HphA-A73N-F	CTGATCGCTGCT***AAT***ACCATGACGGGA	5'
34	HphA-A73N-R	TCCCGTCATGGT***ATT***AGCAGCGATCAG	3'
35	HphA-D157N-F	GGACTCACTGTA***AAC***TTTGCCGCCGAAG	5'
36	HphA-D157N-R	CTTCGGCGGCAAA***GTT***TACAGTGAGTCC	3'
37	HphA-M186F-F	CTCGACCATTTT***TTC***CTCTGTGATACC	5'
38	HphA-M186F-R	GGTATCACAGAG***GAA***AAAATGGTCGAG	3'
39	HphA-H184F-F185C-M186F-F	CCTTATCTCGAC***TTTTGTTTC***CTCTGTGATACC	5'
40	HphA-H184F-F185C-M186F-R	GGTATCACAGAG***GAAACAAAA***GTCGAGATAAGG	3'

Note: The underlined nucleotide sequences are the target of the corresponding restriction enzyme. The bold-italicized nucleotides are the mutation site. Primers #1 and #2 were designed in the previous study on HphA characterization.[6]

References

1. Koketsu, K.; Mitsuhashi, S.; Tabata, K., Identification of homophenylalanine biosynthetic genes from the cyanobacterium Nostoc punctiforme PCC73102 and application to its microbial production by Escherichia coli. *Appl Environ Microbiol* **2013,** *79* (7), 2201-8.
2. Kumar, V. P.; West, A. H.; Cook, P. F., Kinetic and chemical mechanisms of homocitrate synthase from *Thermus thermophilus*. *J Biol Chem* **2011,** *286* (33), 29428-29439.
3. Huisman, F. H.; Koon, N.; Bulloch, E. M.; Baker, H. M.; Baker, E. N.; Squire, C. J.; Parker, E. J., Removal of the C-terminal regulatory domain of alpha-isopropylmalate synthase disrupts functional substrate binding. *Biochemistry* **2012,** *51* (11), 2289-97.
4. Phan, C. S.; Ling, Z.; Mehjabin, J. J.; Matsuda, K.; Prakoso, N. I.; Umezawa, T.; Wakimoto, T.; Okino, T., Doubly Homologated Tyrosine-Containing Peptides from the Cyanobacterium *Microcystis aeruginosa* NIES-4285 and Their Biosynthesis. *J Nat Prod* **2024,** *87* (11), 2629-2639.
5. Corpet, F., Multiple sequence alignment with hierarchical clustering. *Nucleic Acids Res* **1988,** *16* (22), 10881-90.
6. Stewart, L. E.; Owens, S. L.; Ahmed, S. R.; Lang Harman, R. M.; Mori, S., Characterization of HphA: The First Enzyme in the Homologation Pathway of L-Phenylalanine and L-Tyrosine. *ChemBioChem* **2024,** *25* (16), e202400369.
